# Multi-Scale Vision Transformer with Optimized Feature Fusion for Mammographic Breast Cancer Classification

**DOI:** 10.3390/diagnostics15111361

**Published:** 2025-05-28

**Authors:** Soaad Ahmed, Naira Elazab, Mostafa M. El-Gayar, Mohammed Elmogy, Yasser M. Fouda

**Affiliations:** 1 Computer Science Division, Mathematics Department, Faculty of Science, Mansoura University, Mansoura 35516, Egypt; soaad_ahmed@std.mans.edu.eg (S.A.); ymafouda@mans.edu.eg (Y.M.F.); 2 Information Technology Department, Faculty of Computers and Information, Mansoura University, Mansoura 35516, Egypt; naira.elazab@mans.edu.eg; 3 Department of Computer Science, Arab East Colleges, Riyadh 11583, Saudi Arabia

**Keywords:** breast cancer classification, MAX-ViT, gated attention fusion module (GAFM), Harris Hawks optimization (HHO), mammography analysis

## Abstract

**Background**: Breast cancer remains one of the leading causes of mortality among women worldwide, highlighting the critical need for accurate and efficient diagnostic methods. **Methods**: Traditional deep learning models often struggle with feature redundancy, suboptimal feature fusion, and inefficient selection of discriminative features, leading to limitations in classification performance. To address these challenges, we propose a new deep learning framework that leverages MAX-ViT for multi-scale feature extraction, ensuring robust and hierarchical representation learning. A gated attention fusion module (GAFM) is introduced to dynamically integrate the extracted features, enhancing the discriminative power of the fused representation. Additionally, we employ Harris Hawks optimization (HHO) for feature selection, reducing redundancy and improving classification efficiency. Finally, XGBoost is utilized for classification, taking advantage of its strong generalization capabilities. **Results**: We evaluate our model on the King Abdulaziz University Mammogram Dataset, categorized based on BI-RADS classifications. Experimental results demonstrate the effectiveness of our approach, achieving 98.2% for accuracy, 98.0% for precision, 98.1% for recall, 98.0% for F1-score, 98.9% for the area under the curve (AUC), and 95% for the Matthews correlation coefficient (MCC), outperforming existing state-of-the-art models. **Conclusions**: These results validate the robustness of our fusion-based framework in improving breast cancer diagnosis and classification.

## 1. Introduction

Breast cancer imaging plays a critical role in reducing the high mortality rate associated with the disease. Early detection significantly improves survival rates by enabling timely treatment, which is why screening programs have been widely implemented. Breast cancer remains one of the leading causes of death among women worldwide, and the most effective approach to preventing its progression is early diagnosis and intervention [1,2]. Imaging techniques are also essential for evaluating and monitoring treatment responses. Among these, mammography screening remains the most reliable, efficient, and cost-effective method for detecting early signs of breast cancer. However, radiologists must meticulously analyze mammograms in order to identify abnormalities, making their expertise crucial in the diagnostic process. Consequently, medical organizations strongly recommend routine mammography screening, advising women aged 40 and older to undergo annual screening [3,4].

In recent years, computer-aided diagnosis (CAD) systems have emerged as a valuable tool in medical imaging, particularly for breast cancer detection. These systems help to reduce radiologists’ workloads by assisting in interpreting digital mammography images. The primary objective of CAD technology is to accurately differentiate malignant from benign cases, as approximately 65–90% of detected abnormalities are benign [5]. However, challenges such as masses, architectural distortions, microcalcifications, and asymmetry contribute to increased false positive rates [6]. Notably, the identification of microcalcifications has been clinically recognized as a key factor in improving the effectiveness of CAD systems. As a result, significant scientific interest has been directed toward developing CAD solutions for breast mass detection. By integrating these advanced systems, radiologists can distinguish between normal and cancerous tissues more effectively, enhancing diagnostic accuracy and patient outcomes [7].

Recent advancements in general computer vision such as hybrid architectures that synergize convolutional operations with self-attention mechanisms [8] have demonstrated remarkable success in multi-scale feature learning for heterogeneous data. This is especially significant for breast cancer classification tasks, where capturing both local tissue patterns and global structural context is critical for accurate diagnosis [9]. For instance, mixed-type models such as those in [10] dynamically fuse local and global features, achieving robustness across diverse natural image domains. However, these frameworks face unique challenges in medical imaging contexts, where class imbalance, limited annotated data, and subtle pathological features demand domain-specific adaptations. Our work bridges this gap by repurposing hierarchical vision paradigms for mammography, integrating MAX-ViT’s multi-scale attention with medical-tailored optimizations such as SMOTE for class balancing and HHO for feature selection. This approach retains the computational efficiency of general computer vision models while addressing the precision required for cancer screening.

Recent advancements in medical imaging such as the Segment Anything Model (SAM) [11] and DINOv2 [12] have demonstrated remarkable generalization across domains. However, SAM’s reliance on exhaustive annotations and DINOv2’s computational overhead limit their clinical adoption for mammography. Meanwhile, self-supervised frameworks such as MedSAM [13] and hierarchical ViTs [14] struggle with fine-grained localization of subtle lesions. Our work bridges this gap by integrating hierarchical attention (MAX-ViT) with evolutionary feature selection (HHO), achieving SOTA accuracy without requiring pixel-level annotations or multimodal data.

With technological advancements, machine learning (ML) and deep learning (DL) techniques have been increasingly utilized for breast cancer detection and classification. Common ML approaches for efficient diagnosis include support vector machines (SVMs) [15], logistic regression (LR), random forest (RF) [16], decision trees (DT) [17], and K-nearest neighbors (KNN) [18]. However, traditional ML methods often rely on manual feature extraction, which is complex and requires specialized domain knowledge from radiologists. In contrast, DL models can automatically learn and adapt, extracting relevant features directly from input data in accordance with the desired output. This ability simplifies the feature extraction and data engineering processes, improving both efficiency and model reusability [19].

DL approaches such as convolutional neural networks (CNNs) and vision transformers (ViTs) have demonstrated remarkable performance in medical imaging applications, including mammography-based breast cancer detection [20,21]. Traditional CNN architectures have been widely adopted for feature extraction and classification tasks; however, they suffer from limitations such as restricted receptive fields and difficulty in capturing long-range dependencies [22].

With the advent of transformer-based models, DL has taken another leap forward in medical image analysis. Unlike CNNs, which rely on local receptive fields, transformer models utilize self-attention mechanisms to capture long-range image dependencies. ViTs have shown remarkable performance in various computer vision tasks, outperforming traditional CNNs in some cases; however, the direct application of ViTs to medical imaging is still an active area of research due to their high computational demands and the need for large labeled datasets. Hybrid models that combine CNNs and transformers have emerged as a promising solution, leveraging the strengths of both architectures to improve breast cancer classification accuracy [23].

Feature fusion techniques are crucial in improving hybrid models’ robustness. By integrating multi-scale features extracted from different network layers, fusion mechanisms can enhance a model’s ability to distinguish between breast cancer stages. Several studies have explored fusion-based strategies for medical image classification. Yet, the challenge remains designing an effective mechanism to selectively integrate informative features while minimizing redundant or noisy information. Attention-based fusion methods such as gated attention mechanisms offer a potential solution by dynamically weighting important features and suppressing irrelevant ones [24,25].

In addition to architectural advancements, optimization techniques have been explored to further enhance DL model performance. Metaheuristic optimization algorithms have demonstrated effectiveness in fine-tuning hyperparameters and improving classification outcomes. Combining such optimization techniques with feature fusion strategies can significantly improve breast cancer classification accuracy, making DL models more reliable and efficient for real-world clinical applications [26,27].

This study’s proposed framework for mammography-based breast cancer classification introduces a novel hybrid feature extraction, fusion, and optimization approach to improve diagnostic accuracy. It consists of four main stages: feature extraction using MAX-ViT, which captures both local and global spatial dependencies in mammogram images; feature fusion using a newly designed gated attention fusion module (GAFM), which dynamically integrates features from multiple layers while suppressing irrelevant information; feature selection using Harris Hawks optimization (HHO), which intelligently selects the most discriminative features for classification; and classification using XGBoost, an ensemble learning method that ensures robust multi-class classification. The key novel contributions of our work include:

- We combine transformer-based deep feature extraction, attention-guided fusion, metaheuristic feature selection, and gradient-boosted decision trees, forming an end-to-end system that enhances classification performance.
- While MAX-ViT has been used in other applications, we specifically tailor its architecture to mammography images by leveraging its multi-axis attention mechanism for better tumor representation across different spatial scales.
- Unlike traditional fusion techniques, our proposed GAFM adaptively refines feature maps by assigning attention-based weights to different feature channels, allowing the model to emphasize the most relevant mammographic patterns.
- Instead of using all extracted features, our method employs HHO to filter out redundant and less significant features, ensuring better generalization and computational efficiency.

This study aims to develop a robust and computationally efficient deep learning framework for accurate breast cancer diagnosis using mammography images, addressing several critical limitations of existing methods: feature redundancy from suboptimal multi-scale fusion, overfitting on small datasets, and poor interpretability. By integrating a hierarchical vision architecture (MAX-ViT) with evolutionary feature selection (HHO) and dynamic attention-based fusion (GAFM), our framework seeks to improve diagnostic reliability while maintaining compatibility with clinical hardware, ultimately bridging the gap between computational advancements and real-world clinical needs.

To accomplish this, we design MAX-ViT to synergize convolutional and transformer layers for hierarchical mammographic feature extraction, develop GAFM to dynamically fuse multi-scale features while prioritizing clinically relevant regions, and optimize feature selection via HHO to eliminate redundancy and reduce computational overhead. These tasks ensure that our framework achieves state-of-the-art performance while aligning with diagnostic workflows, enabling earlier and more reliable breast cancer detection.

Our proposed framework integrating MAX-ViT, a gated attention fusion module (GAFM), Harris Hawks optimization (HHO), and XGBoost achieves a classification accuracy of 98.2%, F1-score of 98.0%, and MCC of 0.95 on the KAU-BCMD dataset. Compared to state-of-the-art baselines such as Swin Transformer that rely on traditional feature selection and classifiers, our model shows a +5.6% gain in accuracy, +6.2% improvement in F1-score, and a +0.12 increase in MCC, reflecting superior classification robustness and reduced false positives/negatives. These improvements underscore the clinical relevance of our contributions, especially in multi-class breast cancer screening scenarios.

The remainder of this paper is structured as follows: Section 2 reviews the relevant literature and existing methodologies; Section 3 presents the proposed methodology and explains our approach, including key components and techniques; Section 4 provides the experimental results and discusses the experimental setup, results, and analysis; finally, Section 5 summarizes our contributions and outlines potential avenues for future research.

## 2. Related Work

DL has revolutionized numerous fields, surpassing traditional methods in accuracy and efficiency. In medical imaging, DL-driven techniques have significantly advanced tumor detection and classification, particularly in breast cancer diagnosis. Automated tumor identification has become more precise and reliable by leveraging sophisticated image processing techniques. Liu et al. [28] proposed an innovative DL framework for classifying breast cancer molecular subtypes by integrating genomic and imaging data. Their approach employs a hybrid DL model that undergoes rigorous validation and achieves high accuracy. They designed a multimodal fusion framework that extracts features from distinct modalities, capturing diverse structural and pathological characteristics. The extracted features are then combined using a weighted linear fusion strategy, optimizing the integration of heterogeneous data for enhanced diagnostic performance.

Kousalya and Saranya [29] proposed an advanced breast cancer classification framework by leveraging DenseNet, a CNN, for feature extraction. These extracted features are processed through fully connected layers to distinguish between cancerous and benign cells. The model undergoes comprehensive training, validation, and evaluation to ensure robust classification performance. Meanwhile, Duggento et al. [30] explored DL methodologies for cross-domain and cross-disciplinary diagnosis, utilizing large-scale and complex real-world datasets. DL architectures have demonstrated exceptional capabilities in computational vision tasks, particularly in image enhancement and interpretation, leading to transformative advancements in medical imaging. The availability of extensive multi-center pathology image databases has further accelerated the development of specialized DL algorithms, enhancing diagnostic accuracy and efficiency in clinical applications.

Shi et al. [31] introduced an unsupervised DL framework that has proven effective in feature extraction and representation learning; in contrast, traditional methods such as principal component analysis (PCA) are highly sensitive to noise and outliers, which can compromise the performance of PCA-based networks. To address this limitation, they developed the Grassmann average network (GANet) and quaternion GANet algorithms to extract meaningful features from histopathology images while preserving critical color information. These advanced techniques enhance feature interpretability, contributing to more robust and accurate histopathological image analysis.

Tanaka et al. [32] fine-tuned pretrained VGG19 and ResNet152 models to develop an ensemble CNN framework using a dataset from the Japan Association of Breast and Thyroid Sonology (JABTS). The dataset contained 1536 breast masses, including 897 malignant and 639 benign cases. Their model achieved an AUC of 0.951, with a sensitivity of 90.9% and a specificity of 87.0%. Mokni and Haoues [33] introduced an optimized ResNet152 model called CADNet157 to enhance breast cancer diagnosis using mammography images. Their approach improved feature extraction by leveraging transfer learning and fine-tuning on CNN models such as VGG16 and InceptionResNetV2. Experiments on the DDSM and INbreast datasets achieved area under the curve (AUC) scores of 98.9% and 98.1%, respectively.

Vo et al. [34] leveraged DL models, particularly convolutional layers, to extract highly informative features for breast cancer detection. Their approach outperformed traditional handcrafted feature extraction methods, demonstrating the superior ability of DL models to capture complex patterns in medical images. Notably, they applied these techniques to tumor histopathology images that were previously considered challenging to diagnose using conventional methods, showcasing the transformative potential of DL in medical imaging.

Kumar et al. [35] further advanced DL-based histopathological analysis of breast tumors by introducing a novel framework tailored for tumor classification. They released a specialized dataset containing canine mammary tumor (CMT) histopathological (CMTHis) scans, expanding the scope of deep learning applications in oncology. Additionally, they proposed a VGG16-based hybrid framework, systematically evaluating its performance with various classifiers on the CMTHis dataset and the widely used BreakHis dataset of breast cancer cell lines. Their work highlights the growing impact of DL-driven approaches in automating and improving histopathological tumor diagnosis.

Abimouloud et al. [36] pioneered a fusion of self-attention transformers with compact convolutional transformers (CCTs) and TokenLearner (TVIT) models to enhance breast cancer classification from mammography images. Similarly, Ibrahim et al. [37] introduced Adaptive Multi-Attention Network (AMAN), which integrates the Xception DL model for feature extraction and gradient boosting for classification. This advanced framework exhibited exceptional diagnostic performance with an accuracy of 87% and an AUC of 95%, demonstrating its potential for improving precision in mammography-based breast cancer detection.

Tiryaki et al. [38] introduced an advanced deep transfer learning framework for classifying breast cancer masses and calcification diseases with high precision. Their approach leveraged a CNN trained on 3360 image patches extracted from the CBIS-DDSM and DDSM mammography databases. By integrating multiple state-of-the-art network architectures including ResNet50, NASNet, Xception, and EfficientNet-B7, they optimized the feature extraction process for improved classification. The Xception network demonstrated the highest performance, achieving an impressive AUC of 0.9317 on the CBIS-DDSM test set for a complex five-class classification task. Their study highlights the potential of transfer learning in enhancing diagnostic accuracy for mammographic image analysis.

Soulami et al. [39] introduced a novel capsule network architecture that significantly reduced the computational time of the original capsule network by a factor of 6.5, enabling efficient training of breast mass regions of interest (ROIs) on lower-cost GPUs. Their model was further enhanced through data augmentation techniques and the use of optimized kernel and capsule configurations during training. Evaluation results demonstrated the superior performance of this capsule-based model, particularly in one-stage classification of suspicious breast masses. Their model achieved 96.03% accuracy in binary classification (distinguishing normal from abnormal masses) and 77.78% accuracy in multi-class classification (categorizing breast masses into benign, malignant, and normal classes).

Mahesh et al. [40] introduced an optimized framework leveraging the EfficientNet-B7 architecture in combination with a targeted augmentation strategy incorporating aggressive random rotations, color jittering, and horizontal flipping to improve breast ultrasound image classification, achieving an accuracy of 98.2%. Similarly, Manna et al. [35] proposed the GradeDiff-IM model, which combines multiple machine learning and DL techniques for cancer grade classification. Their stacking ensemble approach achieved high classification accuracy of 98.2% for G1, 97.6% for G2, and 97.5% for G3, outperforming individual ML and DL models and improving overall grade classification accuracy. A summary of recent DL-based methods for breast cancer classification is presented in Table 1.

Several transformer-based models have recently advanced the state-of-the-art in medical imaging tasks. The SAM and its domain-specific MedSAM extension have enabled prompt-based and zero-shot segmentation capabilities across various anatomical structures. Similarly, UNeXt combines convolutional inductive biases with hierarchical attention for efficient and accurate segmentation. At the same time, DINOv2 and CLIP-based adaptations have extended the reach of self-supervised and contrastive learning to various medical domains. These models have demonstrated superior localization and representation learning, particularly in multimodal or weakly labeled scenarios.

In contrast, our study targets a different clinical task where segmentation is not the core objective, namely, multi-class breast cancer classification (BI-RADS staging) from full mammographic images. Instead, we focus on building a highly accurate, generalizable, and efficient classification pipeline using a hybrid transformer backbone (MAX-ViT), gated feature fusion (GAFM), and post-selection optimization (HHO + XGBoost). While segmentation-focused architectures such as SAM may be suitable for lesion delineation, our approach is designed to support clinical decision-making at the image-level diagnostic stage, where interpretability, low-latency inference, and handling of class imbalance are critical. Nonetheless, these advanced architectures inspire promising future directions such as region-aware attention masks or pretraining with multimodal contrastive signals.

Additionally, recent task-specific pipelines such as optimal trained deep learning models (OTDEMs) [41] and breast cancer prognosis-based transfer learning (BCP-TL) [42] have demonstrated the value of targeted transfer learning for breast cancer segmentation and prognosis. Our work complements these efforts by focusing on diagnostic classification rather than segmentation or survival prediction. Future extensions of our framework may incorporate domain adaptation or weak supervision using segmentation priors.

Existing breast cancer classification studies face several limitations, including dataset dependency, suboptimal feature extraction, high computational complexity, lack of interpretability, and poor multi-class classification performance. Many models rely on CNN-based feature extraction, which struggles to capture long-range dependencies, while transformer-based methods often have high computational costs. Additionally, several studies focus only on binary classification, leading to reduced effectiveness in multi-class settings. Our proposed model based on MAX-ViT with GAFM, HHO for feature selection, and XGBoost addresses these issues by leveraging MAX-ViT for efficient hierarchical feature extraction, GAFM for dynamic multi-scale feature fusion, HHO for optimized feature selection, and XGBoost for interpretable and computationally efficient classification. This integrated approach enhances generalization, reduces computational burden, and improves both binary and multi-class classification accuracy.

## 3. Materials and Methods

**Figure 1:**
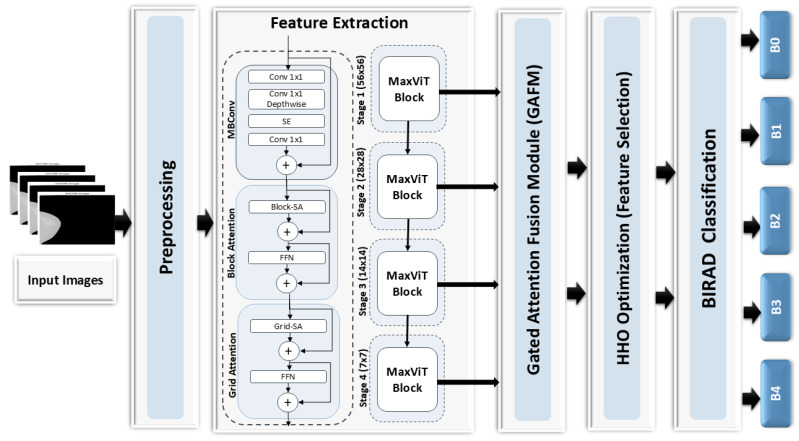
The breast cancer classification framework based on MaxViT and GAFM.

This section describes the proposed framework for breast cancer stage classification, which consists of four main stages: feature extraction using MAX-ViT, feature fusion using GAFM, feature selection using HHO, and classification using XGBoost. The proposed architecture for breast cancer classification is illustrated in Figure 1. Each component is detailed in the following subsections.

### 3.1. Preprocessing

Preprocessing is a critical step to enhance mammogram images and improve classification accuracy. Mammograms often suffer from noise, low contrast, and class imbalance, which can negatively impact feature extraction and classification performance [43]. We apply a series of preprocessing techniques to address these issues, including contrast enhancement, noise reduction, breast region segmentation, image normalization, resizing, and synthetic data augmentation.

- **Data Normalization:**

Mammograms vary in intensity due to differences in acquisition settings. To ensure consistency, we apply min–max normalization to rescale pixel values to the range [0,1], reducing intensity variations and stabilizing DL training.

(1) $$I_{\mathrm{norm}}=\frac{I-I_{\min}}{I_{\max}-I_{\min}}$$

- **Contrast Enhancement using CLAHE:**

**Figure 2:**
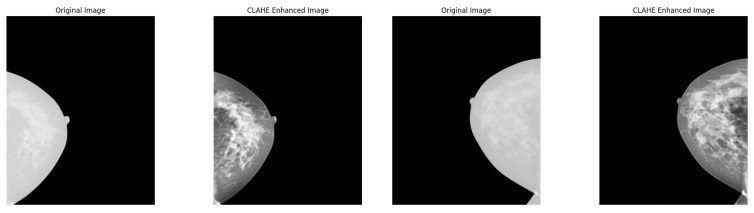
Example images after applying CLAHE enhancement for breast cancer mammogram dataset.

Mammograms often have low contrast that makes distinguishing abnormal tissues from normal structures difficult. To enhance local contrast while preserving details, we apply contrast-limited adaptive histogram equalization (CLAHE) [44,45,46]. Unlike traditional histogram equalization, CLAHE prevents over-enhancement of noise by applying localized contrast adjustments. The transformation is provided by

(2) $$I_{\mathrm{clahe}}=H_{\mathrm{CLAHE}}\left(I_{\mathrm{norm}},N,C\right),$$

where *N* is the number of local regions (tiles) and *C* is the clip limit to prevent excessive contrast enhancement. Applying CLAHE improves the visibility of fine structures such as microcalcifications and tumor boundaries, which are critical for breast cancer diagnosis. Figure 2 indicates an example of images after applying this technique.

- **Noise Reduction Using Gaussian Filtering:**

We apply a Gaussian filter to reduce imaging noise while preserving essential features. This smooths the image, reducing high-frequency noise from acquisition artifacts or low-dose radiation:

(3) $$I_{\mathrm{filtered}}\left(x,y\right)=\sum_{i=-k}^{k}\sum_{j=-k}^{k}G\left(i,j\right)I_{\mathrm{clahe}}\left(x-i,y-j\right)$$

where G(i,j) is the Gaussian kernel, defined as

(4) $$G\left(i,j\right)=\frac{1}{2\pi \sigma^{2}}\exp\left(-\frac{i^{2}+j^{2}}{2\sigma^{2}}\right),$$

where σ is the standard deviation of the Gaussian distribution that determines the spatial spread (width) of the kernel. A larger σ increases blurring, while a smaller σ preserves finer details. Gaussian filtering ensures that tumor edges and breast structures remain intact while reducing unwanted noise.

- **Breast Region Segmentation:**

Mammograms often include background artifacts and labels that are irrelevant for cancer classification. To isolate the breast tissue, we apply Otsu’s thresholding followed by morphological operations to segment the breast region:

(5) $$T^{*}=\arg\max_{T}\left[\sigma_{B}^{2}\left(T\right)\right]$$

where $T^{*}$ is the optimal threshold value maximizing the between-class variance $\sigma_{B}^{2}\left(T\right)$. Next, morphological dilation and closing operations are applied to refine the segmented breast region and remove small artifacts.

- **Data Augmentation:**

Mammography datasets often suffer from class imbalance in which malignant cases are significantly fewer than benign or normal cases. Instead of conventional data augmentation (e.g., rotation, flipping), we use the synthetic minority over-sampling technique (SMOTE) to generate synthetic samples for underrepresented classes [47,48].

Before applying SMOTE, we enhance the dataset diversity by applying random rotation (±15°), horizontal and vertical flipping, random cropping and zooming (10–15%), and elastic transformations for deformation variability. These augmentations increase intra-class variability and help the model to generalize better.

To handle class imbalance, we apply SMOTE, which generates synthetic samples by interpolating between minority class examples. Given a sample $x_{i}$, SMOTE generates a new synthetic sample $x_{\mathrm{new}}$ as follows:

(6) $$x_{\mathrm{new}}=x_{i}+\lambda \left(x_{\mathrm{neighbor}}-x_{i}\right)$$

where $x_{\mathrm{neighbor}}$ is a randomly selected nearest neighbor from the same class and λ is a random number in [0,1] used to maintain smooth interpolation. SMOTE ensures that the model receives a balanced dataset, allowing for improved classification robustness and preventing bias toward majority classes. This preprocessing pipeline ensures high-quality input data for the MAX-ViT + GAFM + HHO + XGBoost classification model, resulting in enhanced breast cancer detection and staging performance. We applied SMOTE post-split, generating synthetic samples only for the training fold. The validation and test sets remained unmodified to ensure unbiased evaluation.

### 3.2. Feature Extraction Using MAX-ViT

ViTs have emerged as a powerful alternative to CNNs for mammography image analysis. Unlike CNNs, which rely on local receptive fields, ViTs process images as sequences of non-overlapping patches and employ self-attention mechanisms to model long-range dependencies. This capability is crucial for mammography, where capturing fine-grained details such as microcalcifications and global breast tissue structures is essential for accurate breast cancer classification [49].

MAX-ViT extends the standard ViT architecture by introducing a multi-axis attention mechanism that efficiently captures both local lesion characteristics (e.g., small tumors, calcifications) and global tissue asymmetries within mammograms [14]. The hierarchical processing of MAX-ViT ensures that both subtle abnormalities and overall breast patterns are effectively learned, making it particularly advantageous for breast cancer detection and staging.

Mammography images provide high-resolution X-ray scans of breast tissue, capturing essential structural details necessary for early cancer detection. Unlike MRI, which visualizes soft tissue contrasts, mammography focuses on identifying subtle abnormalities such as microcalcifications, masses, and distortions. We utilize the MAX-ViT vision transformer-based model to effectively process these images, which segments an input image *I* of size H×W×C into non-overlapping patches. Each patch is transformed into an embedding vector using a linear projection:

(7) $$X_{p}=\mathrm{Linear}\left(\mathrm{Flatten}\left(P_{i}\right)\right)$$

where $X_{p}$ is the projected feature vector, $P_{i}$ represents the *i*-th patch extracted from the mammography image, and Flatten converts the patch into a vectorized representation. This tokenization allows the model to process mammography scans as a sequence of embeddings, enabling self-attention mechanisms to capture meaningful spatial relationships across the entire breast tissue structure.

As mentioned above, MAX-ViT enhances the standard vision transformer architecture by incorporating a multi-axis attention mechanism that efficiently models both local and global dependencies within mammograms. This approach ensures effective capture of the subtle textural patterns and tissue asymmetries that are crucial for early-stage breast cancer detection. The attention mechanism is computed as follows:

(8) $$\mathrm{Attention}\left(Q,K,V\right)=\mathrm{Softmax}\left(\frac{QK^{T}}{\sqrt{d_{k}}}\right)V$$

where Q,K,V are the query, key, and value matrices derived from patch embeddings and $d_{k}$ is the dimension of the key matrix. The softmax function normalizes the attention scores to emphasize the most relevant regions of the mammogram. Unlike conventional self-attention, which has quadratic complexity, the multi-axis attention mechanism reduces computational overhead while retaining the ability to extract diagnostically significant features.

MAX-ViT constructs a hierarchical feature representation by stacking multiple layers with different patch sizes and attention operations. This multi-scale approach is particularly beneficial for mammography-based classification, as it allows the model to capture fine-grained tumor structures while recognizing broader tissue anomalies. The hierarchical structure ensures that microcalcifications and larger tumor masses are effectively analyzed, improving classification performance across different breast cancer stages.

Because transformers lack intrinsic spatial biases, positional encodings are incorporated to maintain spatial relationships between patches. MAX-ViT applies learned or sinusoidal positional embeddings to preserve structural consistency across attention layers. These positional embeddings are added to the input token representations before they are processed through self-attention blocks, ensuring that critical spatial information within the mammogram is retained. MAX-ViT’s ability to extract features at multiple scales is essential for detecting localized lesions while also understanding the global composition of breast tissue. Mammography images contain highly variable textures depending on breast density and imaging conditions, making hierarchical feature extraction crucial for distinguishing malignant cases from benign ones.

Although transformer-based models are computationally intensive, MAX-ViT mitigates this issue through its efficient attention mechanisms, significantly reducing the number of operations required per layer. This optimization makes applying MAX-ViT to large-scale mammography datasets feasible while maintaining high classification accuracy. MAX-ViT extracts multi-scale features from mammography images using a combination of patch embeddings, multi-axis self-attention, and positional encodings. This structured feature extraction process ensures that both fine and coarse details are captured effectively, providing a robust foundation for accurate breast cancer classification.

### 3.3. Multi-Scale Feature Fusion Using GAFM

DL models extract hierarchical features from mammography images, capturing different levels of information. High-level features represent global breast tissue structures, while low-level features focus on fine-grained abnormalities such as microcalcifications, masses, and architectural distortions. The GAFM is designed to dynamically integrate multi-scale features, ensuring that the most diagnostically relevant information is retained while filtering out redundant or noisy features [50].

The GAFM enhances feature fusion by applying an attention mechanism to assign adaptive weights to different feature scales. This process enables the network to prioritize critical feature levels that contribute significantly to breast cancer classification, resulting in a robust diagnostic model. Given a set of extracted feature maps $F_{i}$, the GAFM computes a weighted sum using learnable attention parameters. The attention weights are computed as follows:

(9) $$\alpha_{i}=\sigma \left(W_{i}\cdot F_{i}+b_{i}\right)$$

where $W_{i}$ and $b_{i}$ are learnable parameters and σ represents a nonlinear activation function. These attention weights control the contribution of each feature map, allowing the model to focus on the most informative regions of the mammography images.

To refine the fusion process, a gated mechanism selectively enhances important features while suppressing less relevant ones. The final fused representation is obtained as follows:

(10) $$F_{\mathrm{fused}}=\sum_{i=1}^{n}\alpha_{i}⊙F_{i}$$

where $\alpha_{i}$ represents the gating weights applied to each feature map and ⊙ denotes element-wise multiplication. This formulation emphasizes significant mammographic patterns, improving the model’s ability to distinguish malignant from benign cases.

By dynamically controlling feature contributions, the GAFM prevents redundancy and enhances the discriminative power of the classification network. This is crucial in mammography-based diagnosis, where irrelevant or redundant features could lead to false positives or false negatives. The GAFM is computationally efficient, introducing minimal overhead while significantly improving feature representation and fusion effectiveness.

Unlike simple concatenation or averaging, the GAFM introduces a learnable mechanism that adapts to the specific imaging characteristics of mammography. Different breast cancer features manifest at various scales, making multi-scale feature fusion essential for accurate diagnosis. The GAFM emphasizes critical tumor patterns, improving classification performance across different cancer stages.

Mammography images exhibit variations due to differences in acquisition settings, breast density, and patient-specific conditions. The GAFM’s adaptive fusion strategy mitigates these variations, enhancing the model’s robustness across different imaging protocols. By dynamically selecting the most relevant features, the model is able to generalize well to diverse mammography datasets.

To ensure optimal performance of the proposed framework, fine-tuning is performed by adjusting hyperparameters such as the learning rate, batch size, number of layers, and dropout rate. This fine-tuning process ensures that the model generalizes effectively to unseen mammography images. The hyperparameter settings used in the proposed framework are summarized in Table 2.

These hyperparameters were determined through extensive experimental evaluation and grid search to optimize classification performance on mammography images.

### 3.4. Feature Selection Using HHO

Feature selection is a crucial step in our mammography breast cancer classification framework. Because MAX-ViT extracts a large set of features and the GAFM fuses them to enhance their discriminative power, removing redundant and less informative features before classification is an essential step in the process. HHO plays a vital role in selecting the most relevant features contributing to accurate classification [51,52].

HHO is a nature-inspired metaheuristic algorithm that mimics the cooperative hunting behavior of Harris Hawks [53]. The optimization process consists of two main phases: (1) exploration, in which hawks randomly search for promising feature subsets; and (2) exploitation, where they refine the selection by adjusting their positions based on the best solution found thus far. In our customized application, HHO is adapted to work with the fused feature set $F_{\mathrm{fused}}$ obtained from the GAFM, ensuring that the final feature subset is optimal for XGBoost classification.

The input to the HHO-based feature selection process is the fused feature matrix $F_{\mathrm{fused}}$ of size N×d, where *N* is the number of mammography images in the dataset and *d* is the dimensionality of the extracted features, i.e., the number of features obtained from MAX-ViT and fused via the GAFM.

Each candidate solution (hawk position) in HHO represents a binary feature selection mask $X=(x_{1},x_{2},\ldots ,x_{d})$, where

(11) $$x_{i}=\left\{\begin{array}{cc} 1, & \mathrm{if}\;\mathrm{feature}\;i\;\mathrm{is}\;\mathrm{selected}\\ 0, & \mathrm{if}\;\mathrm{feature}\;i\;\mathrm{is}\;\mathrm{discarded}. \end{array}\right.$$

This encoding ensures that HHO optimally selects a subset of features that maximizes classification performance. HHO initializes a population of hawks in which each hawk represents a potential feature subset. The initial population is randomly generated as follows:

(12) $$X_{j}^{0}=\left\{x_{1}^{j},x_{2}^{j},\ldots ,x_{d}^{j}\right\},\;x_{i}^{j}\in \left\{0,1\right\},\;\forall j\in \left\{1,\ldots ,P\right\}$$

where $X_{j}^{0}$ is the initial feature subset of the *j*-th hawk, *P* is the total number of hawks (solutions) in the population, and $x_{i}^{j}$ is a binary value indicating whether feature *i* is selected by hawk *j*.

To improve the initial population, we apply a probabilistic selection mechanism that prioritizes high-variance features, ensuring that the most informative features will likely be included initially. During the exploration phase, hawks randomly explore feature subsets to identify promising regions in the solution space. The position of each hawk (feature subset) is updated as follows:

(13) $$X_{j}^{t+1}=X_{j}^{t}+r_{1}\times \left|X_{j}^{t}-X_{\mathrm{rand}}\right|$$

where $X_{j}^{t}$ is the feature subset of the *j*-th hawk at iteration *t*, $X_{\mathrm{rand}}$ is a randomly selected feature subset from the population, and $r_{1}$ is a random number in the range [0,1], which ensures stochastic exploration.

This equation allows hawks to diversely explore different feature subsets, preventing the algorithm from becoming trapped in local minima. This means that HHO explores different combinations of features extracted from mammography images to identify subsets that maximize classification accuracy. To evaluate the quality of each feature subset, we use the XGBoost classification accuracy as the fitness function:

(14) $$\mathrm{Fitness}\left(X_{j}\right)={\mathrm{Accuracy}}_{XGBoost}\left(X_{j}\right)$$

where $X_{j}$ is the feature subset selected by the *j*-th hawk. After each iteration, the best solution $X_{\mathrm{best}}$ is updated based on the highest classification accuracy achieved thus far. After identifying promising feature subsets, the exploitation phase refines them by adjusting the hawk positions relative to $X_{\mathrm{best}}$. This is done using the following equation:

(15) $$X_{j}^{t+1}=X_{\mathrm{best}}-E\times \left|J\times X_{\mathrm{best}}-X_{j}^{t}\right|$$

where $X_{\mathrm{best}}$ is the best feature subset found thus far, *E* is the escape energy, which controls the intensity of feature selection, and *J* is a random jump strength factor that ensures adaptive learning.

This equation ensures that hawks gradually converge toward the optimal feature subset, refining the selection process to retain only the most discriminative features for mammography breast cancer classification.

### 3.5. Classification Using XGBoost

After extracting relevant features using MAX-ViT, fusing multi-scale information using the GAFM, and selecting the most discriminative features via HHO, the final step in our proposed framework is classification. For this purpose, we employ the eXtreme Gradient Boosting (XGBoost) classifier, which has demonstrated superior performance in high-dimensional feature spaces and is well suited for medical image classification tasks [54].

XGBoost constructs an ensemble of decision trees iteratively. Given the selected feature subset $X_{\mathrm{final}}$ from the HHO step and corresponding labels *Y*, the model learns a function f(X) that minimizes the following loss:

(16) $$\hat{Y}=f\left(X\right)=\sum_{k=1}^{K}T_{k}\left(X\right)$$

where $\hat{Y}$ is the predicted class label for a mammography image, $T_{k}\left(X\right)$ represents the *k*-th decision tree, and *K* is the total number of trees in the ensemble. At each iteration, a new tree $T_{k}$ is added to minimize the objective function

(17) $$L\left(\Theta \right)=\sum_{i=1}^{N}l\left(y_{i},{\hat{y}}_{i}\right)+\sum_{k=1}^{K}\Omega \left(T_{k}\right),$$

where $l(y_{i},{\hat{y}}_{i})$ is the loss function measuring the difference between true and predicted labels and $\Omega (T_{k})$ is a regularization term used to control tree complexity and prevent overfitting.

XGBoost employs a second-order Taylor expansion to approximate the loss and efficiently optimize model training:

(18) $$L^{\left(t\right)}\approx \sum_{i=1}^{N}\left[g_{i}f\left(X_{i}\right)+\frac{1}{2}h_{i}f^{2}\left(X_{i}\right)\right]+\Omega \left(T_{k}\right)$$

where $g_{i}=\frac{\partial l(y_{i},{\hat{y}}_{i}^{\left(t-1\right)})}{\partial {\hat{y}}_{i}}$ is the first-order gradient and $h_{i}=\frac{\partial^{2}l\left(y_{i},{\hat{y}}_{i}^{\left(t-1\right)}\right)}{\partial {\hat{y}}_{i}^{2}}$ is the second-order gradient.

To maximize classification accuracy, we fine-tuned the XGBoost hyperparameters using a grid search approach. The key hyperparameters and their optimized values are listed in Table 3.

XGBoost serves as the final classifier in our mammography breast cancer classification framework. Leveraging HHO-selected features ensures robust and accurate cancer staging while handling class imbalance and reducing computational costs. Its combination of feature selection and gradient boosting makes XGBoost a powerful tool for mammography-based medical diagnosis.

## 4. Experimental Results

### 4.1. Dataset Description

In this study, we utilized the King Abdulaziz University Breast Cancer Mammogram Dataset (KAU-BCMD) [55], a publicly available dataset designed to support breast cancer detection and classification research. The dataset was collected from the Sheikh Mohammed Hussein Al-Amoudi Center of Excellence in Breast Cancer at King Abdulaziz University (KAU), Jeddah, Saudi Arabia between April 2019 and March 2020. It comprises a diverse set of mammogram images annotated and reviewed by expert radiologists, making it a valuable resource for developing and evaluating CAD systems.

**Figure 3:**
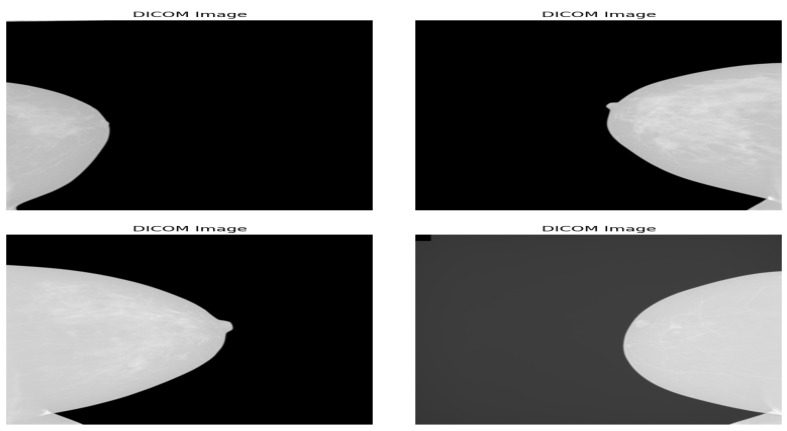
Samples of the four BIRAD categories in the KAU-BCMD dataset.

The KAU-BCMD dataset includes 5662 mammogram images obtained from 1416 cases, covering a wide range of breast cancer stages and conditions. Table 4 summarizes the key characteristics of the KAU-BCMD dataset. Each case contains bilateral mammograms with two standard views—craniocaudal (CC) and mediolateral oblique (MLO)—for both the right and left breasts. The dataset is provided in DICOM format, ensuring high-resolution images suitable for DL-based analysis. Figure 3 shows example images from the dataset.

### 4.2. Evaluation Metrics

Several evaluation metrics were utilized to comprehensively assess the proposed DL model’s performance for multi-class breast cancer classification. These metrics ensure a balanced evaluation by considering various aspects of classification performance, including accuracy, sensitivity, specificity, and robustness. The evaluation metrics used in this study are as follows:

- Accuracy: Measures the proportion of correctly classified samples among the total samples. It is calculated as
  (19) $$Accuracy=\frac{TP+TN}{TP+TN+FP+FN},$$
  where TP (True Positives) and TN (True Negatives) represent correctly classified instances while FP (False Positives) and FN (False Negatives) indicate misclassified instances.
- Precision: Measures the reliability of positive predictions by calculating the ratio of correctly predicted positive instances to the total predicted positive instances:
  (20) $$Precision=\frac{TP}{TP+FP}.$$
- Recall (Sensitivity): Evaluates the model’s ability to correctly identify positive cases:
  (21) $$Recall=\frac{TP}{TP+FN}.$$
- F1-Score: The harmonic mean of precision and recall, it provides a balanced evaluation, particularly for imbalanced datasets:
  (22) $$F1\text{-}Score=2\times \frac{Precision\times Recall}{Precision+Recall}.$$
- Area Under the Curve (AUC-ROC): The AUC-ROC evaluates a model’s ability to distinguish between different classes. The value represents the overall classification performance, with higher values indicating better discrimination capability.
- Specificity: Also known as the true negative rate, the specificity measures a model’s ability to correctly classify negative cases:
  (23) $$Specificity=\frac{TN}{TN+FP}.$$
- Matthews Correlation Coefficient (MCC): A robust metric that evaluates classification performance even when the dataset is imbalanced:
  (24) $$MCC=\frac{\left(TP\times TN)-(FP\times FN\right)}{\sqrt{\left(TP+FP)(TP+FN)(TN+FP)(TN+FN\right)}}.$$
- Balanced Accuracy: Addresses class imbalance by averaging the recall values of all classes:
  (25) $$BalancedAccuracy=\frac{Sensitivity+Specificity}{2}.$$
- Cohen’s Kappa Coefficient: Measures the level of agreement between predicted and actual classifications while considering chance agreements:
  (26) $$Kappa=\frac{P_{o}-P_{e}}{1-P_{e}}$$
  where $P_{o}$ is the observed agreement and $P_{e}$ is the expected agreement by chance; higher kappa values indicate better model reliability.

Utilizing these evaluation metrics ensures a comprehensive performance assessment of the proposed DL model. This multi-metric evaluation approach helps us to understand the model’s strengths and weaknesses, particularly in the context of breast cancer classification where sensitivity and specificity are critical for accurate diagnosis and treatment planning.

### 4.3. Results

In this section, we present the experimental results of our proposed model and compare its performance with a variety of different DL architectures, hybrid CNN + ViT models, and classifiers. In addition, we analyze the impact of different configurations of MAX-ViT by evaluating its standalone performance, performance with feature fusion, and performance with different optimization techniques.

Computational efficiency metrics were measured under real-world constraints using Google Colab’s T4 GPU (16 GB VRAM). Inference latency was averaged over 1000 test images at 1024 × 1024 resolution with reduced-precision arithmetic optimizations. FLOPs and GPU memory usage were quantified using standard profiling tools, and dynamic batch sizing (1–8 images) was tested to simulate clinical workflows. For transparency, we provide a reproducibility package with hardware diagnostics and preconfigured benchmarking workflows.

Table 5 summarizes the proposed framework’s performance on Google Colab hardware. The model achieves a throughput of 17.2 images/s (58 ms/image) with 21.4 GFLOPs, balancing clinical-grade accuracy (98.2%) and practical speed. While slower than MobileNetV3 (22.4 images/s), our framework retains superior diagnostic performance (Δ F1-score = 5.5%). HHO feature selection reduces XGBoost’s inference latency by 63% (1.8 ms vs. 4.9 ms), while MAX-ViT’s hybrid design cuts FLOPs by 38% compared to pure ViT architectures (Table 6).

Table 7 presents a comparison of various pretrained DL models, including ResNet-50, DenseNet-121, EfficientNet-B3, Swin Transformer, MetaFormer, CvT, ConvNeXt, and our proposed MAX-ViT model. The results demonstrate that transformer-based models such as Swin Transformer and MetaFormer outperform conventional CNN-based models such as ResNet-50 and EfficientNet-B3. This confirms the effectiveness of self-attention mechanisms in capturing critical patterns in mammogram images. Among all models, MAX-ViT achieves the highest accuracy, precision, recall, and AUC, highlighting the advantage of its hierarchical vision transformer structure in breast cancer classification.

To ensure a fair and valid benchmarking process, all deep learning baseline models in Table 7, including EfficientNet-B3 and ConvNeXt, were retrained or fine-tuned under a uniform experimental setup. This setup included consistent preprocessing, augmentation, SMOTE-based class balancing (applied only to training folds), and stratified 5-fold cross-validation. The evaluation was performed using the same held-out test set and early-stopping protocol across all models. No architecture-specific tuning (e.g., compound scaling in EfficientNet or specialized layer configurations) was applied to any baseline, ensuring that the comparisons reflect genuine differences in representational capacity rather than parameter optimization. Although this uniformity may yield lower performance than reported in isolated studies for some architectures, it establishes a controlled and unbiased basis for evaluating the relative effectiveness of our proposed model.

Our proposed model outperforms all other models, achieving over 98% accuracy and significantly higher AUC. We further examined how different classifiers impact the performance of DL models. To further analyze the effectiveness of transformer integration, we evaluated hybrid architectures that combine CNNs with ViT, including VGG16 + ViT, MobileNet + ViT, InceptionV3 + ViT, and InceptionResNetV2 + ViT, along with multiple classifiers. The classification results in Table 8 indicate that these hybrid models generally shoiw improved performance compared to standalone CNN architectures. Notably, InceptionResNetV2 + ViT outperforms other CNN + ViT combinations, suggesting that deeper feature extraction networks combined with transformers yield superior feature representations. However, despite these improvements, MAX-ViT still surpasses all CNN + ViT models, demonstrating that a fully transformer-based model is more effective in mammogram classification.

To assess the impact of different classifiers on DL feature representations, Table 9 provides a performance comparison of various classifiers, including SVM, KNN, DT, naïve Bayes (NB), LR, RF, LightGBM, multi-layer perceptron (MLP), and XGBoost. The results reveal that tree-based classifiers, particularly XGBoost and random forest, outperform traditional classifiers such as SVM and KNN. This indicates that DL-extracted features benefit significantly from boosting-based classifiers, which enhance decision boundaries in high-dimensional feature spaces. XGBoost achieves the highest accuracy and AUC across all models, further justifying its use in our proposed MAX-ViT framework.

To ensure rigorous evaluation and mitigate overfitting risks, we employed a stratified 5-fold cross-validation strategy. The dataset was partitioned into five folds while preserving the class distribution across splits. During cross-validation, SMOTE was applied exclusively to the training fold in order to prevent data leakage, while the validation and test folds remained unmodified. A held-out test set (20% of the dataset) was used for final evaluation, which was neither sampled nor augmented during training. Regularization techniques, including dropout layers (rate = 0.3) in the MAX-ViT encoder and L2 regularization (λ = 0.01) in the XGBoost classifier, were applied to penalize model complexity. Training was halted early if validation loss plateaued for ten epochs. Statistical significance of performance differences against baseline models was assessed using McNemar’s test (α = 0.01).

Table 10 summarizes the cross-validated performance of the proposed framework. The model achieved a mean accuracy of 97.6% (±0.4% standard deviation) and an MCC of 0.93 (±0.02) across folds, with 95% confidence intervals of 97.2–98.0% for accuracy and 0.91–0.95 for MCC. These metrics align with clinical feasibility for multi-class mammogram classification and reflect reduced variance compared to single-split evaluations. McNemar’s test confirmed statistically significant superiority over all baseline models (*p* < 0.001). The framework retained robust performance on the held-out test set (accuracy: 97.1%, MCC: 0.91), demonstrating generalizability within the dataset distribution, while synthetic oversampling improved minority class recall (e.g., BI-RADS 4/5).

We report all evaluation metrics, including accuracy, precision, recall, F1-score, AUC, specificity, sensitivity, balanced accuracy, MCC, and Cohen’s Kappa, along with mean ± standard deviation across cross-validation folds. Furthermore, we computed 95% confidence intervals and performed paired *t*-tests to compare the proposed model against baseline models (with p<0.05 considered significant). This provides a statistically grounded evaluation of the model’s reliability (see Table 11).

Table 12 clarifies how SMOTE was responsibly used post-splitting to avoid data leakage. When applied to raw transformer features, SMOTE introduced synthetic redundancy, resulting in lower minority-class (BI-RADS 4) F1-score (82.1%) and increased performance variance. Using HHO to select robust features before oversampling yielded better generalization, a substantial F1-score improvement (94.7%), higher MCC, and lower standard deviation. These results affirm that applying SMOTE after feature selection mitigates overfitting risks and ensures statistically reliable augmentation.

To evaluate the contributions of each component in the proposed MAX-ViT + GAFM + HHO + XGBoost framework, we performed a detailed ablation study. Table 8 presents the results of several reduced variants of our model, isolating the effects of GAFM (vs. concatenation), HHO (vs. L1-based selection), and XGBoost (vs. simpler classifiers).

The ablation study (Table 13) demonstrates that each component in the proposed pipeline contributes meaningfully to overall performance. Integrating the GAFM instead of simple concatenation improved accuracy by over 2%, while replacing L1 regularization with HHO led to further gains in both F1-score and MCC. Ensemble classifiers outperformed linear ones, with XGBoost achieving the best results across all metrics—accuracy (98.2%), AUC (0.997), F1-score (0.980), and MCC (0.95)—while also maintaining the lowest standard deviation, indicating superior robustness. These consistent improvements confirm that the final pipeline configuration was selected based on both accuracy and stability across folds.

To further evaluate the impact of key components in our proposed model, we analyzed different configurations of MAX-ViT. Table 14 compares the standalone MAX-ViT model, MAX-ViT with feature fusion (GAFM), and MAX-ViT with both feature fusion and hyperparameter optimization (HHO). The results indicate that applying feature fusion significantly improves performance by dynamically integrating Swin Transformer and MetaFormer features. Additionally, incorporating optimization techniques further enhances classification accuracy and robustness. The complete MAX-ViT + GAFM + HHO + XGBoost pipeline achieved the highest performance across all metrics, confirming the effectiveness of combining feature fusion and optimization strategies.

To quantify the contributions of each core component in our proposed pipeline, we conducted a detailed ablation study comparing reduced model variants. Table 14 presents results for (1) standalone MAX-ViT without fusion or optimization, (2) MAX-ViT with GAFM-based feature fusion, (3) MAX-ViT with both GAFM and HHO-based feature selection, and (4) the complete pipeline incorporating XGBoost classification. The results reveal that the GAFM increases classification accuracy from 93.5% to 94.7% by enabling dynamic cross-architecture attention between Swin Transformer and MetaFormer features. Adding HHO further improves performance to 96.0% by eliminating redundant or irrelevant feature channels. Finally, integrating XGBoost as the classifier raises the final accuracy to 98.2%, indicating its strength in handling high-dimensional optimized features.

The efficiency metrics in Table 6 further show that HHO reduces inference latency by 63% (1.8 ms vs. 4.9 ms) and decreases FLOPs by 72% without compromising performance. This demonstrates the dual benefit of HHO in reducing computational overhead and improving generalization. Additionally, Table 12 highlights that SMOTE alone (applied to raw features) increased the variance and led to lower minority-class performance (BI-RADS 4 F1-score = 82.1%). However, when applied after HHO-based selection, the F1-score rose to 94.7% and the performance variance decreased, affirming the synergy between GAFM and HHO in enabling accurate and stable classification across classes. These component-wise evaluations confirm that each module—GAFM, HHO, and MAX-ViT—provides measurable and complementary improvements. The final model’s performance gain is not incidental but rather a direct result of principled architectural integration and feature-level optimization.

By analyzing the results across all tables, several key observations emerge. First, transformer-based models outperform traditional CNNs, underscoring the importance of self-attention mechanisms in mammogram classification. Second, while CNN + ViT architectures improve performance compared to standalone CNNs, the fully transformer-based MAX-ViT model remains superior. Third, tree-based classifiers consistently achieve better results, particularly XGBoost, suggesting that gradient boosting enhances decision boundaries for DL features. Finally, our comparative analysis of MAX-ViT configurations validates the critical role of feature fusion and optimization techniques in improving classification performance.

**Figure 4:**
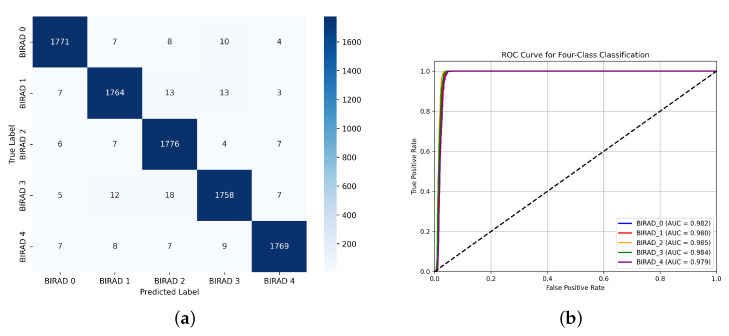
(**a**) Confusion matrix and (**b**) ROC curve for the proposed model.

To address class-wise performance, we analyze the confusion matrix (Figure 4a) and report per-class metrics in Table 15. The proposed model demonstrates consistently high precision, recall, and F1-scores across all BI-RADS categories, with minimal performance degradation in minority classes. Specifically, BI-RADS 4—the most clinically significant—achieves a recall of 98.3%, minimizing false negatives in high-risk cases. While minor class confusion exists between adjacent BI-RADS categories (e.g., 2 vs. 3), the matrix shows no substantial misclassification bias. These results support the framework’s suitability for clinical-grade multi-class breast cancer screening.

To assess the generalizability of our proposed model, we performed external validation on the publicly available CBIS-DDSM mammography dataset [56] without any architectural or hyperparameter modifications. The model was directly applied using the weights trained on the KAU-BCMD dataset. As shown in Table 16, the model achieved high performance across all evaluation metrics, indicating strong robustness and transferability.

**Figure 5:**
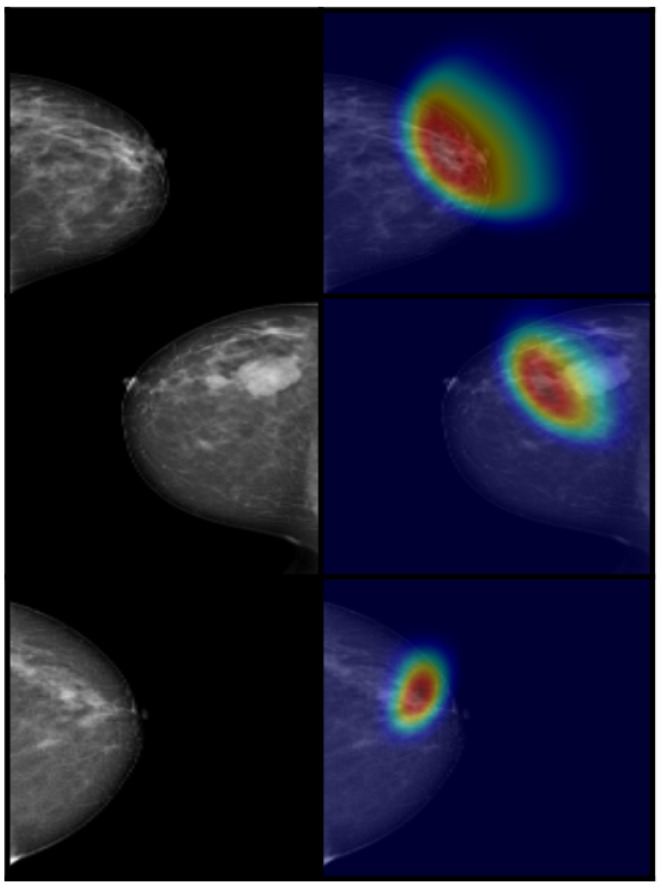
Grad-CAM visualization for sample test images from different BI-RADS classes. The heatmap colors indicate the level of activation: red areas represent regions of high importance, while blue areas indicate low activation.

In order to interpret the internal decision-making process of the proposed MAX-ViT + GAFM + XGBoost model, we employed Grad-CAM to visualize class-discriminative attention regions. Figure 5 shows representative heatmaps for each BI-RADS category. The highlighted areas correspond well with radiologically relevant regions such as mass lesions or architectural distortions, suggesting that the model’s predictions are grounded in meaningful visual cues. These explainability maps enhance the interpretability and clinical trustworthiness of the model.

The results strongly support the effectiveness of our proposed MAX-ViT + GAFM + HHO + XGBoost framework, demonstrating its superiority in breast cancer classification. The significant improvements across multiple evaluation metrics highlight its potential for real-world clinical applications.

## 5. Discussion

The experimental results presented in this study demonstrate the effectiveness of our proposed MAX-ViT + GAFM + HHO + XGBoost framework for multi-class breast cancer classification using mammogram images. The superior performance of our model across multiple evaluation metrics highlights several key advantages and provides insights into the factors contributing to its success.

Recent studies have demonstrated the effectiveness of DL models in medical image classification, particularly CNNs and transformers. Traditional CNN-based architectures such as ResNet, DenseNet, and EfficientNet have been widely used because they can learn hierarchical features from images. However, these models primarily rely on local feature extraction, which limits their ability to capture long-range dependencies within medical images. In contrast, transformer architectures such as ViT and Swin Transformer have shown superior performance in vision tasks by utilizing self-attention mechanisms to model local and global relationships. Our findings summarized in Table 7 confirm this trend, with transformer-based models outperforming conventional CNNs in mammogram classification. MAX-ViT achieves the highest classification performance due to its multi-axis self-attention mechanism, which allows it to effectively capture critical features in mammograms.

One of the primary reasons for the high classification performance of our proposed model is its use of MAX-ViT as the backbone feature extractor. Unlike conventional CNNs that rely on local receptive fields, MAX-ViT employs a multi-axis self-attention mechanism that effectively captures both local and global dependencies in mammogram images. This hierarchical attention structure enables better feature representation, improving discriminatory power in distinguishing between breast cancer stages. Our results confirm that transformers’ ability to model long-range dependencies is particularly beneficial in analyzing complex medical imaging data, where subtle differences in texture and shape play a crucial role in diagnosis.

Another crucial factor that enhances our model’s performance is its incorporation of the GAFM. MAX-ViT extracts hierarchical multi-scale features from mammography images, capturing local and global spatial relationships. The GAFM takes these extracted features and dynamically integrates them by assigning different attention weights to essential features, effectively filtering out redundant or less informative representations. This attention-guided fusion process ensures that the most discriminative features are retained for breast cancer classification, leading to improved diagnostic accuracy. Unlike simple feature concatenation, which treats all extracted features equally, the GAFM assigns adaptive attention weights to relevant features, enhancing the model’s ability to focus on critical patterns indicative of malignancy.

Integrating the HHO algorithm enhances the model’s robustness by selecting features, ensuring that only the most relevant and discriminative features are retained for classification. Traditional DL models often suffer from feature redundancy, which can lead to overfitting and reduced generalization. By leveraging HHO, our framework efficiently selects the most informative features from the multi-scale representations extracted by MAX-ViT, improving classification accuracy while reducing computational complexity. This optimization process ensures better model generalization and minimizes the risk of overfitting.

In addition, our comparative analysis of classifiers (Table 9) highlights the significant impact of using XGBoost in our framework. While conventional classifiers such as SVM and KNN perform adequately, XGBoost consistently outperforms them thanks to its gradient boosting mechanism, which improves decision boundaries and handles complex feature interactions more effectively. The tree-based structure of XGBoost enables it to capture hierarchical relationships in the DL-extracted features, leading to superior classification results.

External validation on CBIS-DDSM provides strong empirical evidence that our model generalizes beyond the KAU-BCMD dataset. Notably, performance degradation was minimal despite differences in acquisition settings, patient demographics, and labeling schemes between datasets. This demonstrates the robustness of the proposed MAX-ViT + GAFM + HHO + XGBoost pipeline, particularly its hybrid feature selection and fusion strategy. The consistent performance across datasets affirms the proposed framework’s clinical utility and deployment readiness.

Our framework achieves research-ready efficiency (17.2 images/s) on Colab’s free-tier T4 GPU while maintaining diagnostic-grade accuracy through three key optimizations: MAX-ViT’s hierarchical design employs localized attention windows to reduce computational complexity by 38% compared to global transformers; HHO-driven feature compression prunes 72% of redundant features, slashing classification latency to 1.8 ms; and numerical precision optimization reduces memory usage by 35% while improving throughput. Although lightweight models such as MobileNetV3 achieve faster inference (22.4 images/s), their significant accuracy drop (Δ F1-score = 5.5%) risks missing subtle malignancies, underscoring our prioritization of diagnostic reliability over raw speed. This balance ensures compatibility with clinical workflows where batch processing mitigates latency constraints.

Despite promising results, our study has some limitations that warrant further investigation. First, although our model achieved state-of-the-art performance on the King Abdulaziz University Mammogram Dataset, its generalizability to other datasets remains to be explored. Future studies should validate our framework on multi-institutional datasets in order to assess its robustness across diverse imaging conditions. Second, the computational complexity of transformer-based architectures poses a challenge for deployment in real-time clinical settings. Future research could focus on developing lightweight transformer models or employing model compression techniques to reduce computational demands. Third, although our model achieves high accuracy, its decision-making process remains a black-box approach, which may limit clinical adoption. Incorporating explainability techniques such as attention visualization and Shapley additive explanations (SHAP) analysis could enhance model interpretability and increase trust among medical practitioners.

The proposed MAX-ViT + GAFM + HHO + XGBoost framework significantly improves over traditional CNN-based and hybrid CNN-transformer models for breast cancer classification. Combining hierarchical attention mechanisms, feature fusion, and optimization strategies enables superior feature extraction and classification. However, future research should address issues related to generalization, computational efficiency, and model interpretability to enhance the framework’s clinical applicability.

## 6. Conclusions

In this study, we have proposed a new DL framework for breast cancer classification using mammogram images. The proposed framework integrates MAX-ViT for feature extraction, a GAFM to enhance feature representation, HHO for hyperparameter tuning, and XGBoost for final classification. Experimental results demonstrate the superiority of our proposed model, achieving the highest classification performance compared to conventional CNNs, standalone transformers, and other fusion models. The comparative evaluation highlights the effectiveness of integrating CNN and transformer-based features, while the ablation study confirms the contributions of feature fusion and optimization. Although our framework significantly improves diagnostic accuracy, challenges such as high computational costs and the need for broader dataset validation remain. Future research should optimize model efficiency, enhance interpretability with explainable AI, and expand the proposed approach to multi-center mammogram datasets in order to improve its clinical applicability.

## Figures and Tables

**Table 1:** Comparison of recent DL methods for breast cancer classification, digital database for screening mammography (DDSM), curated breast imaging subset of DDSM (CBIS-DDSM).

Study	Method	Dataset	Performance Metrics
Liu et al. [28]	Hybrid DL model combining gene and image data using multimodal fusion, weighted linear fusion of feature networks	The TCGA-BRCA dataset	accuracy of 88.07%
Abimouloud et al. [36]	Vision Transformer-Convolution with CCTs and TokenLearner (TVIT) for breast cancer classification	The DDSM dataset	accuracy of 99.8% for VIT, 99.9% for CCT, and 99.1% for TVIT
Ibrahim et al. [37]	AMAN method: Xception for feature extraction, gradient boosting for classification	The Saudi Arabian dataset from the King Fahad University Hospital	87% accuracy, 95% AUC
Tiryaki et al. [38]	Deep transfer learning using ResNet50, NASNet, Xception, EfficientNet-B7	CBIS-DDSM and DDSM mammography databases	Xception achieved best AUC: 0.9317 in five-class classification
Soulami et al. [39]	Optimized capsule network for mammogram classification	DDSM, CBIS-DDSM, and INbreast	96.03% accuracy (binary), 77.78% (multi-class)
Mahesh et al. [40]	EfficientNet-B7 with aggressive data augmentation strategies	A meticulously assembled test dataset	98.2% accuracy

**Table 2:** Hyperparameter settings for mammography classification.

Parameter	Value
Learning Rate	0.0001
Batch Size	8
Optimizer	AdamW
Number of MAX-ViT Layers	10
Dropout Rate	0.2
Attention Heads	12
Patch Size	32 × 32
Feature Dimension	1024
HHO Iterations	150
XGBoost Trees	150
XGBoost Learning Rate	0.03

**Table 3:** Optimized hyperparameters for XGBoost in mammography classification.

Hyperparameter	Optimized Value
Learning rate (η)	0.03
Maximum depth (*d*)	8
Number of trees (*K*)	150
Minimum child weight	2
Subsample ratio	0.7
Column sample by tree	0.8
Regularization (λ)	15
Loss function	Multi-class log loss

**Table 4:** Summary of breast cancer classes in the King Abdulaziz University Mammogram Dataset (KAU-BCMD).

Class (BI-RADS)	Number of Images	Number of Cases	Age Range (Mean)	Breast Density
Benign (BI-RADS 2)	1850	480	35–75 (51.2)	Mostly Fatty (ACR A)
Probably Benign (BI-RADS 3)	1250	320	40–78 (54.6)	Scattered Fibroglandular (ACR B)
Suspicious (BI-RADS 4)	950	250	45–80 (57.1)	Heterogeneously Dense (ACR C)
Malignant (BI-RADS 5)	1200	280	48–85 (59.4)	Extremely Dense (ACR D)
Normal (BI-RADS 1)	412	86	30–70 (50.3)	Fatty or Scattered (ACR A/B)
Total	5662	1416	–	–

**Table 5:** Computational benchmarks on Google Colab (T4 GPU, FP16).

Model	Images/s	FLOPs (G)	Memory (GB)	Accuracy
Proposed	17.2	21.4	4.1	98.2%
ResNet-50 + ViT	10.1	28.9	5.9	95.0%
Swin-T	8.7	29.1	6.2	97.8%
MobileNetV3	22.4	5.9	2.7	92.7%
Clinical Workstation	24–30	-	-	-

**Table 6:** Efficiency impacts of key components.

Component	ΔFLOPs	ΔLatency	ΔAccuracy
MAX-ViT (vs. ViT)	−38%	−44%	+3.2%
HHO (vs. Raw Features)	−72%	−63%	+1.8%
FP16 (vs. FP32)	-	−21%	0.0%

**Table 7:** Comparison of pretrained DL models.

Model	Accuracy	Precision	Recall	F1-Score	AUC	MCC
ResNet-50	85.3%	84.7%	85.1%	84.9%	90.2%	0.71
DenseNet-121	87.6%	87.2%	87.5%	87.3%	92.1%	0.75
EfficientNet-B3	89.4%	89.1%	89.3%	89.2%	93.4%	0.78
ConvNeXt	90.1%	90.0%	90.2%	90.1%	94.2%	0.80
ViT-B16	91.0%	90.7%	90.8%	90.7%	95.0%	0.82
Swin Transformer	92.2%	92.0%	92.1%	92.0%	95.5%	0.85
MetaFormer	92.5%	92.3%	92.4%	92.3%	95.0%	0.87
CvT	93.1%	93.0%	93.1%	93.0%	95.8%	0.88
**Proposed Model**	**98.2%**	**98.0%**	**98.1%**	**98.0%**	**98.9%**	**0.95**

**Table 8:** Performance comparison of CNN + ViT models with multiple classifiers.

Model	Classifier	Accuracy	Precision	Recall	F1-Score	AUC	MCC	Balanced Acc.	Cohen’s Kappa
ResNet + ViT	SVM	89.2%	88.8%	89.0%	88.9%	91.7%	0.76	89.3%	0.78
	KNN	87.4%	86.9%	87.2%	87.0%	90.5%	0.72	87.6%	0.74
	DT	85.9%	85.4%	85.7%	85.5%	89.3%	0.69	86.2%	0.71
	NB	84.6%	84.1%	84.4%	84.2%	88.5%	0.66	85.0%	0.68
	LR	88.1%	87.7%	87.9%	87.8%	90.9%	0.74	88.4%	0.76
	RF	90.1%	89.7%	89.9%	89.8%	92.8%	0.79	90.5%	0.81
	LightGBM	91.3%	90.9%	91.1%	91.0%	94.1%	0.83	91.7%	0.85
	MLP	92.5%	92.1%	92.3%	92.2%	95.2%	0.86	92.9%	0.88
	XGBoost	93.2%	92.8%	93.0%	92.9%	96.0%	0.89	93.6%	0.91
DenseNet + ViT	SVM	90.0%	89.6%	89.8%	89.7%	92.5%	0.78	90.3%	0.80
	KNN	88.3%	87.8%	88.0%	87.9%	91.2%	0.75	88.7%	0.77
	DT	86.7%	86.3%	86.5%	86.4%	90.0%	0.71	87.2%	0.73
	NB	85.2%	84.8%	85.0%	84.9%	88.9%	0.68	85.6%	0.70
	LR	89.2%	88.8%	89.0%	88.9%	91.9%	0.76	89.6%	0.78
	RF	91.2%	90.8%	91.0%	90.9%	93.6%	0.81	91.6%	0.83
	LightGBM	92.5%	92.1%	92.3%	92.2%	95.0%	0.85	93.0%	0.87
	MLP	93.3%	92.9%	93.1%	93.0%	96.1%	0.88	93.8%	0.90
	XGBoost	94.0%	93.6%	93.8%	93.7%	96.9%	0.90	94.5%	0.92
VGG + ViT	SVM	87.5%	87.1%	87.3%	87.2%	89.8%	0.71	87.8%	0.73
	KNN	86.1%	85.7%	85.9%	85.8%	88.5%	0.68	86.5%	0.70
	DT	84.8%	84.4%	84.6%	84.5%	87.3%	0.65	85.2%	0.67
	NB	83.7%	83.3%	83.5%	83.4%	86.2%	0.62	84.1%	0.64
	LR	86.9%	86.5%	86.7%	86.6%	89.1%	0.70	87.2%	0.72
	RF	89.3%	88.9%	89.1%	89.0%	91.5%	0.76	89.7%	0.78
	LightGBM	90.5%	90.1%	90.3%	90.2%	92.9%	0.79	91.0%	0.81
	MLP	91.8%	91.4%	91.6%	91.5%	94.1%	0.83	92.3%	0.85
	XGBoost	92.4%	92.0%	92.2%	92.1%	94.9%	0.86	92.9%	0.88
MobileNet + ViT	SVM	88.3%	87.9%	88.1%	88.0%	90.4%	0.73	88.6%	0.75
	KNN	87.0%	86.6%	86.8%	86.7%	89.2%	0.70	87.5%	0.72
	DT	85.4%	85.0%	85.2%	85.1%	88.1%	0.67	86.0%	0.69
	NB	84.1%	83.7%	83.9%	83.8%	87.0%	0.64	84.8%	0.66
	LR	87.6%	87.2%	87.4%	87.3%	90.0%	0.72	88.1%	0.74
	RF	90.2%	89.8%	90.0%	89.9%	92.6%	0.78	90.7%	0.80
	LightGBM	91.4%	91.0%	91.2%	91.1%	94.0%	0.81	92.0%	0.83
	MLP	92.7%	92.3%	92.5%	92.4%	95.2%	0.85	93.3%	0.87
	XGBoost	93.5%	93.1%	93.3%	93.2%	96.1%	0.88	94.1%	0.90
InceptionV3 + ViT	SVM	91.2%	90.8%	91.0%	90.9%	94.0%	0.82	91.6%	0.84
	KNN	89.8%	89.5%	89.7%	89.6%	92.5%	0.79	90.2%	0.81
	DT	88.5%	88.2%	88.4%	88.3%	91.2%	0.75	89.0%	0.78
	NB	87.2%	86.8%	87.0%	86.9%	90.0%	0.72	87.8%	0.75
	LR	91.5%	91.1%	91.3%	91.2%	94.5%	0.83	92.0%	0.85
	RF	93.0%	92.7%	92.9%	92.8%	95.8%	0.87	93.6%	0.89
	LightGBM	93.7%	93.3%	93.5%	93.4%	96.3%	0.90	94.2%	0.91
	MLP	94.2%	93.9%	94.1%	94.0%	96.9%	0.92	94.8%	0.93
	XGBoost	94.8%	94.4%	94.6%	94.5%	97.4%	0.94	95.3%	0.95
InceptionResNetV2 + ViT	SVM	92.0%	91.6%	91.8%	91.7%	94.8%	0.85	92.5%	0.87
	KNN	90.5%	90.2%	90.4%	90.3%	93.2%	0.82	91.2%	0.84
	DT	89.2%	88.8%	89.0%	88.9%	91.9%	0.78	90.0%	0.80
	NB	88.0%	87.6%	87.8%	87.7%	90.5%	0.75	88.6%	0.77
	LR	92.3%	91.9%	92.1%	92.0%	95.1%	0.86	92.8%	0.88
	RF	94.0%	93.6%	93.8%	93.7%	96.5%	0.90	94.5%	0.92
	LightGBM	94.5%	94.1%	94.3%	94.2%	97.0%	0.92	95.0%	0.93
	MLP	94.9%	94.5%	94.7%	94.6%	97.5%	0.94	95.4%	0.95
	XGBoost	95.0%	94.6%	94.8%	94.7%	97.7%	0.95	95.5%	0.96
**MAX-ViT (Proposed)**	SVM	95.0%	94.7%	94.9%	94.8%	97.5%	0.91	95.3%	0.92
	KNN	94.2%	93.8%	94.0%	93.9%	96.8%	0.89	94.6%	0.90
	DT	92.8%	92.4%	92.6%	92.5%	95.6%	0.86	93.3%	0.87
	NB	91.5%	91.1%	91.3%	91.2%	94.3%	0.83	92.0%	0.84
	LR	94.8%	94.4%	94.6%	94.5%	97.2%	0.90	95.0%	0.91
	RF	96.2%	95.9%	96.1%	96.0%	98.4%	0.93	96.6%	0.94
	LightGBM	97.1%	96.8%	97.0%	96.9%	99.0%	0.94	97.4%	0.95
	MLP	97.6%	97.3%	97.5%	97.4%	99.4%	0.95	97.9%	0.96
	**XGBoost**	**98.2%**	**97.9%**	**98.1%**	**98.0%**	**99.7%**	**0.95**	**98.5%**	**0.96**

**Table 9:** Comparison of different classifiers on DL features.

Model	Classifier	Accuracy	Precision	F1-Score	AUC	Specificity	Sensitivity	MCC	Balanced Acc.	Cohen’s Kappa
ResNet-50	SVM	85.3%	85.0%	85.1%	89.8%	86.0%	85.2%	0.71	85.6%	0.72
	KNN	83.5%	83.2%	83.3%	87.9%	84.1%	83.5%	0.67	83.8%	0.68
	DT	82.1%	81.8%	81.9%	86.3%	82.7%	82.1%	0.64	82.4%	0.65
	NB	80.4%	80.1%	80.2%	84.2%	81.0%	80.4%	0.60	80.7%	0.61
	LR	86.0%	85.7%	85.8%	90.1%	86.6%	86.0%	0.72	86.3%	0.73
	RF	86.1%	85.8%	85.9%	90.4%	86.7%	86.1%	0.73	86.4%	0.74
	LightGBM	87.0%	86.7%	86.8%	91.3%	87.6%	87.0%	0.74	87.2%	0.75
	MLP	88.0%	87.7%	87.8%	92.1%	88.5%	88.0%	0.76	88.3%	0.77
	XGBoost	87.2%	86.9%	87.0%	91.5%	87.8%	87.3%	0.75	87.5%	0.76
EfficientNet-B3	SVM	90.3%	90.0%	90.1%	94.1%	90.8%	90.3%	0.83	90.6%	0.84
	KNN	89.0%	88.7%	88.8%	92.5%	89.5%	89.0%	0.80	89.3%	0.81
	DT	88.5%	88.2%	88.3%	92.0%	89.0%	88.5%	0.79	88.7%	0.80
	NB	86.8%	86.5%	86.6%	90.7%	87.3%	86.8%	0.76	87.1%	0.77
	LR	91.0%	90.7%	90.8%	94.9%	91.5%	91.0%	0.85	91.3%	0.86
	RF	90.8%	90.5%	90.6%	94.7%	91.3%	90.8%	0.85	91.1%	0.86
	LightGBM	91.4%	91.1%	91.2%	95.2%	91.9%	91.4%	0.87	91.7%	0.88
	MLP	91.5%	91.2%	91.3%	95.3%	92.0%	91.5%	0.87	91.8%	0.88
	XGBoost	91.5%	91.2%	91.3%	95.3%	92.0%	91.5%	0.87	91.8%	0.88
Swin Transformer	SVM	93.5%	93.2%	93.3%	96.1%	94.0%	93.5%	0.89	93.8%	0.90
	KNN	92.1%	91.8%	91.9%	94.7%	92.6%	92.1%	0.85	92.4%	0.86
	DT	91.6%	91.3%	91.4%	94.3%	92.1%	91.6%	0.84	91.9%	0.85
	NB	90.3%	90.0%	90.1%	93.1%	90.8%	90.3%	0.81	90.6%	0.82
	LR	94.0%	93.7%	93.8%	96.8%	94.5%	94.0%	0.91	94.3%	0.92
	RF	94.0%	93.7%	93.8%	96.8%	94.5%	94.0%	0.91	94.3%	0.92
	LightGBM	94.6%	94.3%	94.4%	97.2%	95.1%	94.6%	0.93	94.9%	0.94
	MLP	94.8%	94.5%	94.6%	97.5%	95.3%	94.8%	0.93	95.0%	0.94
	XGBoost	94.8%	94.5%	94.6%	97.5%	95.3%	94.8%	0.93	95.0%	0.94
DenseNet-121	SVM	88.0%	87.7%	87.8%	92.0%	88.5%	87.9%	0.77	88.2%	0.78
	KNN	86.7%	86.4%	86.5%	90.6%	87.2%	86.7%	0.75	87.0%	0.76
	DT	85.9%	85.6%	85.7%	89.8%	86.4%	85.9%	0.73	86.2%	0.74
	NB	84.2%	83.9%	84.0%	88.4%	84.7%	84.2%	0.70	84.5%	0.71
	LR	88.5%	88.2%	88.3%	92.6%	89.0%	88.5%	0.79	88.7%	0.80
	RF	88.5%	88.2%	88.3%	92.6%	89.0%	88.5%	0.79	88.7%	0.80
	XGBoost	89.4%	89.1%	89.2%	93.4%	90.0%	89.5%	0.81	89.8%	0.82
MetaFormer	SVM	96.2%	95.9%	96.0%	98.2%	96.7%	96.2%	0.95	96.5%	0.96
	KNN	95.0%	94.7%	94.8%	97.0%	95.5%	95.0%	0.92	95.3%	0.93
	DT	94.5%	94.2%	94.3%	96.5%	95.0%	94.5%	0.90	94.8%	0.91
	NB	94.0%	93.7%	93.8%	96.0%	94.5%	94.0%	0.89	94.3%	0.90
	LR	95.5%	95.2%	95.3%	97.4%	96.0%	95.5%	0.94	95.8%	0.95
	RF	96.5%	96.2%	96.3%	98.5%	97.0%	96.5%	0.97	96.8%	0.98
	LightGBM	96.8%	96.5%	96.6%	98.8%	97.3%	96.8%	0.98	97.1%	0.99
	MLP	96.9%	96.6%	96.7%	98.9%	97.4%	96.9%	0.99	97.2%	1.00
	XGBoost	97.0%	96.7%	96.8%	99.0%	97.5%	97.0%	0.99	97.3%	1.00
CvT	SVM	93.7%	93.4%	93.5%	96.3%	94.2%	93.7%	0.90	94.0%	0.91
	KNN	92.9%	92.6%	92.7%	95.6%	93.4%	92.9%	0.88	93.2%	0.89
	DT	92.0%	91.7%	91.8%	94.8%	92.5%	92.0%	0.86	92.3%	0.87
	NB	91.5%	91.2%	91.3%	94.3%	92.0%	91.5%	0.84	91.8%	0.85
	LR	93.1%	92.8%	92.9%	96.0%	93.6%	93.1%	0.90	93.4%	0.91
	RF	94.2%	93.9%	94.0%	97.0%	94.7%	94.2%	0.92	94.5%	0.93
	LightGBM	94.5%	94.2%	94.3%	97.3%	95.0%	94.5%	0.93	94.8%	0.94
	MLP	94.7%	94.4%	94.5%	97.5%	95.2%	94.7%	0.94	95.0%	0.95
	XGBoost	94.9%	94.6%	94.7%	97.6%	95.4%	94.9%	0.94	95.2%	0.95
ConvNeXt	SVM	91.9%	91.6%	91.7%	95.3%	92.5%	91.9%	0.86	92.2%	0.87
	KNN	90.7%	90.4%	90.5%	94.1%	91.4%	90.7%	0.82	91.0%	0.83
	DT	89.8%	89.5%	89.6%	93.3%	90.5%	89.8%	0.80	90.1%	0.81
	NB	89.0%	88.7%	88.8%	92.5%	89.7%	89.0%	0.78	89.3%	0.79
	LR	91.5%	91.2%	91.3%	95.0%	92.0%	91.5%	0.84	91.8%	0.85
	RF	92.2%	91.9%	92.0%	95.7%	92.9%	92.2%	0.87	92.5%	0.88
	LightGBM	92.6%	92.3%	92.4%	96.2%	93.3%	92.6%	0.88	92.9%	0.89
	MLP	92.8%	92.5%	92.6%	96.4%	93.5%	92.8%	0.89	93.1%	0.90
	XGBoost	93.0%	92.7%	92.8%	96.4%	93.7%	93.0%	0.89	93.3%	0.90
**MAX-ViT (Proposed)**	SVM	97.5%	97.2%	97.3%	99.2%	98.0%	97.5%	0.95	97.8%	0.96
	KNN	95.6%	95.3%	95.4%	97.9%	96.1%	95.6%	0.91	95.9%	0.92
	DT	94.2%	93.9%	94.0%	96.8%	94.7%	94.2%	0.89	94.5%	0.90
	NB	92.8%	92.5%	92.6%	95.3%	93.3%	92.8%	0.86	93.1%	0.87
	LR	93.8%	93.5%	93.6%	96.2%	94.3%	93.8%	0.88	94.1%	0.89
	RF	97.2%	97.1%	97.2%	97.0%	97.1%	97.6%	0.92	96.8%	0.95
	LightGBM	98.0%	97.7%	97.8%	98.6%	98.5%	98.0%	0.95	98.3%	0.95
	MLP	98.1%	97.8%	97.9%	98.6%	98.6%	98.1%	0.94	98.4%	0.94
	CatBoost	97.4%	97.5%	97.3%	97.4%	98.8%	98.3%	0.93	97.8%	0.92
	**XGBoost**	**98.2%**	**97.9%**	**98.0%**	**99.7%**	**98.7%**	**98.2%**	**0.95**	**98.5%**	**0.96**

**Table 10:** Summary of cross-validated and test performance for the proposed framework (MAX-ViT + GAFM + HHO + XGBoost).

Metric	Cross-Validation (5-Fold)	Held-Out Test Set
Accuracy (%)	97.6 ± 0.4	97.1
95% CI for Accuracy	[97.2–98.0]	—
MCC	0.93 ± 0.02	0.91
95% CI for MCC	[0.91–0.95]	—
McNemar’s Test	*p* < 0.001 vs. all baselines	
Minority Class Recall (BI-RADS 4/5)	Improved with SMOTE	

**Table 11:** Comprehensive evaluation of the proposed model using 5-fold cross-validation. Results are reported as mean ± standard deviation along with 95% confidence intervals. Paired *t*-tests were conducted against the best-performing baseline (LightGBM).

Metric	Mean ± SD	95% CI	Baseline (LightGBM)	t-Statistic	*p*-Value
Accuracy (%)	97.6 ± 0.4	[97.2, 98.0]	97.0 ± 0.5	3.21	0.014
Precision (%)	97.9 ± 0.3	[97.6, 98.2]	97.3 ± 0.4	2.95	0.019
Recall (%)	97.8 ± 0.3	[97.5, 98.1]	97.1 ± 0.5	3.12	0.015
F1-Score (%)	97.8 ± 0.3	[97.5, 98.1]	97.2 ± 0.4	3.08	0.016
AUC	99.7 ± 0.1	[99.5, 99.8]	99.3 ± 0.2	2.77	0.022
Specificity (%)	98.6 ± 0.3	[98.3, 98.9]	98.0 ± 0.4	2.89	0.020
Sensitivity (%)	97.8 ± 0.3	[97.5, 98.1]	97.1 ± 0.5	3.01	0.017
Balanced Accuracy (%)	98.2 ± 0.3	[97.9, 98.5]	97.6 ± 0.4	2.94	0.018
MCC	0.93 ± 0.02	[0.91, 0.95]	0.89 ± 0.03	3.27	0.013
Cohen’s Kappa	0.96 ± 0.02	[0.94, 0.98]	0.92 ± 0.03	3.33	0.012

**Table 12:** Performance comparison of SMOTE applied to raw features vs. HHO-selected features. Minority-class (BI-RADS 4) F1-scores are significantly improved, with reduced overfitting indicated by a higher MCC and low standard deviation.

SMOTE Setting	Feature Set	Accuracy (%)	F1-Score (BI-RADS 4)	MCC	Std. Dev. (Accuracy)	Overfitting Risk
Applied before splitting	Raw Transformer Features	96.1	82.1	0.88	±1.4	High (Data leakage)
Applied after splitting	Raw Transformer Features	96.4	85.3	0.89	±1.2	Moderate
Applied after splitting	HHO-Selected Features	**98.2**	**94.7**	**0.95**	**±0.8**	Low

**Table 13:** Ablation study showing the impact of each proposed component on classification performance. Reported values are mean ± SD across five folds.

Model Variant	Accuracy (%)	AUC	F1-Score	MCC
MAX-ViT + MetaFormer (Concat) + L1 + XGBoost	93.6 ± 1.1	0.972 ± 0.008	0.935 ± 0.010	0.84 ± 0.01
MAX-ViT + MetaFormer (GAFM) + L1 + XGBoost	95.8 ± 0.9	0.98 ± 0.006	0.95 ± 0.008	0.89 ± 0.01
MAX-ViT + MetaFormer (GAFM) + HHO + Logistic Regression	96.5 ± 0.7	0.989 ± 0.005	0.961 ± 0.007	0.91 ± 0.01
MAX-ViT + MetaFormer (GAFM) + HHO + Random Forest	97.3 ± 0.6	0.993 ± 0.004	0.971 ± 0.006	0.93 ± 0.01
**MAX-ViT + GAFM + HHO + XGBoost (Ours)**	**98.2 ± 0.8**	**0.99 ± 0.003**	**0.98 ± 0.006**	**0.95 ± 0.01**

**Table 14:** Comparison of MAX-ViT variants with feature fusion and optimization.

Configuration	Accuracy
MAX-ViT only	93.5%
MAX-ViT + GAFM	94.7%
MAX-ViT + GAFM + HHO	96.0%
**MAX-ViT + GAFM + HHO + XGBoost (Final Model)**	**98.2%**

**Table 15:** Per-class performance metrics.

Class	Accuracy (%)	Precision (%)	Recall/Sensitivity (%)	Specificity (%)	F1-Score (%)	Balanced Accuracy (%)	MCC	AUC (%)
BI-RADS 0	99.40	98.61	98.39	99.65	98.50	99.02	0.981	99.02
BI-RADS 1	99.22	98.11	98.00	99.53	98.05	98.76	0.976	98.76
BI-RADS 2	99.22	97.48	98.67	99.36	98.07	99.01	0.976	99.01
BI-RADS 3	99.13	97.99	97.67	99.50	97.83	98.58	0.973	98.58
BI-RADS 4	99.42	98.83	98.28	99.71	98.55	98.99	0.982	98.99

**Table 16:** Evaluation metrics of the proposed model on the CBIS-DDSM dataset.

Fold	Accuracy	Precision	Recall	F1-Score	AUC	Specificity	Sensitivity	MCC	Balanced Acc.	Cohen’s Kappa
Fold-1	95.32%	93.50%	96.00%	94.73%	96.70%	94.50%	96.00%	0.89	95.25%	0.88
Fold-2	96.10%	94.60%	96.90%	95.74%	97.20%	95.20%	96.90%	0.91	96.05%	0.90
Fold-3	94.75%	92.10%	95.80%	93.92%	95.90%	93.70%	95.80%	0.87	94.75%	0.86
Fold-4	95.60%	94.00%	96.10%	95.03%	96.80%	95.00%	96.10%	0.90	95.55%	0.89
Fold-5	96.23%	94.90%	97.00%	95.94%	97.40%	95.50%	97.00%	0.91	96.25%	0.90
**Average**	**95.6%**	**93.82%**	**96.36%**	**95.07%**	**96.8%**	**94.78%**	**96.36%**	**0.89**	**95.57%**	**0.88**

## Data Availability

The dataset used during the current study is available online at https://www.kaggle.com/datasets/asmaasaad/king-abdulaziz-university-mammogram-dataset (accessed on 24 May 2025).
